# COVID-19-Associated Rhino-Orbital Mucormycosis: Histological and Electron Microscopy Characteristics

**DOI:** 10.3390/diagnostics14040429

**Published:** 2024-02-15

**Authors:** Ionuț Isaia Jeican, Delia Ioana Horhat, Mihai Dumitru, Adrian Florea, Lucian Barbu-Tudoran, Bogdan-Alexandru Gheban, Vlad Anton, Corneliu Toader, Maria Aluaș, Costel Vasile Siserman, Nicolae Balica, Daniela Vrînceanu, Silviu Albu

**Affiliations:** 1 Department of Anatomy and Embryology, Iuliu Hatieganu University of Medicine and Pharmacy, 400006 Cluj-Napoca, Romania; jeican.ionut@umfcluj.ro; 2 Department of Ear-Nose-Throat, Victor Babes University of Medicine and Pharmacy Timisoara, 300041 Timisoara, Romania; horhat.ioana@umft.ro (D.I.H.); balica@umft.ro (N.B.); 3 Department of Ear-Nose-Throat, Carol Davila University of Medicine and Pharmacy, 050472 Bucharest, Romania; mihai.dumitru@umfcd.ro (M.D.); daniela.vrinceaunu@umfcd.ro (D.V.); 4 Department of Cell and Molecular Biology, Iuliu Hatieganu University of Medicine and Pharmacy, 400349 Cluj-Napoca, Romania; aflorea@umfcluj.ro; 5 Electron Microscopy Laboratory Prof. C. Crăciun, Faculty of Biology and Geology, Babes-Bolyai University, 400006 Cluj-Napoca, Romania; lucian.barbu@ubbcluj.ro; 6 Electron Microscopy Integrated Laboratory, National Institute for R&D of Isotopic and Molecular Technologies, 400293 Cluj-Napoca, Romania; 7 Department of Histology, Iuliu Hatieganu University of Medicine and Pharmacy, 400349 Cluj-Napoca, Romania; gheban.bogdan@umfcluj.ro; 8 Department of Pathology, Emergency Clinical County Hospital, 400347 Cluj-Napoca, Romania; 9 Department of Medical Biochemistry, Iuliu Hatieganu University of Medicine and Pharmacy, 400349 Cluj-Napoca, Romania; vlad.anton@elearn.umfcluj.ro; 10 Clinic of Neurosurgery, National Institute of Neurology and Neurovascular Diseases, 041914 Bucharest, Romania; corneliu.toader@umfcd.ro; 11 Department of Oral Health, Iuliu Hatieganu University of Medicine and Pharmacy, 400012 Cluj-Napoca, Romania; 12 Institute of Legal Medicine, 400006 Cluj-Napoca, Romania; costel.siserman@elearn.umfcluj.ro; 13 Department of Legal Medicine, Iuliu Hatieganu University of Medicine and Pharmacy, 400006 Cluj-Napoca, Romania; 14 Department of Head and Neck Surgery and Otorhinolaryngology, University Clinical Hospital of Railway Company, Iuliu Hatieganu University of Medicine and Pharmacy, 400015 Cluj-Napoca, Romania; albu.silviu@umfcluj.ro

**Keywords:** mucormycosis, COVID-19, histopathology, scanning electron microscopy, transmission electron microscopy

## Abstract

COVID-19-associated rhino-orbital mucormycosis has become a new clinical entity. This study’s aim was to evaluate the histopathological and ultramicroscopic morphological aspects of this fungal infection. This was an observational retrospective study on eight patients from three tertiary centers in Romania. The tissue samples collected during functional endoscopic sinus surgery were studied through histopathological examination, scanning electron microscopy, and transmission electron microscopy. In the histopathological examination, the morphological aspects characteristic of mucormycosis in all cases were identified: wide aseptate hyphae with right-angle ramifications, which invade blood vessels. One case presented perineural invasion into the perineural lymphatics. And in another case, mucormycosis–aspergillosis fungal coinfection was identified. Through scanning electron microscopy, long hyphae on the surface of the mucosa surrounded by cells belonging to the local immune system were identified in all samples, and bacterial biofilms were identified in half of the samples. Through transmission electron microscopy, aseptate hyphae and bacterial elements were identified in the majority of the samples. Rhino-orbital-cerebral mucormycosis associated with COVID-19 produces nasal sinus dysbiosis, which favors the appearance of bacterial biofilms. The way in which the infection develops depends on the interaction of the fungi with cells of the immune system.

## 1. Introduction

Mucormycosis, also known as zygomycosis, is a fungal infection caused by ubiquitous organisms from the class of *Zygomycetes*, the order of *Mucorales*, most commonly by the genus *Rhizopus* [1]. The epidemiology, pathogenesis, clinical presentation, diagnosis, and management of mucormycosis have all been reviewed extensively, both independently [1,2] and in the context of COVID-19 [3,4,5,6,7]. These fungi are typical environmental organisms but can cause a devastating opportunistic infection, especially for immunosuppressed patients. 

COVID-19-associated rhino-orbital mucormycosis (CAM) is a new clinical entity that reached epidemic proportions in India during the second wave of the COVID-19 pandemic, both because of the humid climate and occupations (the disease was more commonly reported in farmers) [8,9,10,11,12,13]. 

There are several clinical forms of mucormycosis: rhinocerebral, pulmonary, gastrointestinal, cutaneous, and encephalic [14]. Rhino-orbital-cerebral mucormycosis, the most common type of mucormycosis, is a life-threatening infection with mortality rates ranging from 25% to 62% of cases [1]. This is caused by the inhalation of its filamentous (hyphal form) fungi and the subsequent spreading of the fungus to the nose and paranasal sinuses [15]. The infection spreads rapidly from the paranasal sinuses to the orbit, cavernous sinus, and brain, and if it is not diagnosed in time, it leads to death [16]. 

The clinical diagnosis of rhino-orbital-cerebral mucormycosis is based on the identification of the following aspects: necrotic turbinates, blood in nasal discharge along with ipsilateral pain, peri-orbital or peri-nasal swelling with discoloration and induration, ptosis, proptosis or ophthalmoplegia, and, ultimately, multiple cranial nerve palsies [7]. Macroscopic fungal growth on affected tissue has a white, fluffy appearance [17], but in the later stages of infection, affected tissues may become necrotic and black [18]. Tissue biopsies are the clinical specimens of choice and should undergo histopathological examination, which can be accompanied by microbiological examination from specimens of involved sites [19,20]. 

A histopathological examination reveals broad, ribbon-like hyphae with irregular branching invading blood vessels. To perform an accurate differential diagnosis, one should evaluate morphology, width, branching angle, and septation [1,14,21]. To the best of our knowledge, this is the first study evaluating the electron microscopy aspects in CAM.

This study’s aim was to describe and evaluate the histopathological and ultramicroscopic morphological aspects (scanning electron microscopy and transmission electron microscopy) of CAM in a multicenter cohort of patients from Romania.

## 2. Materials and Methods

### 2.1. Ethical and Legal Considerations 

Our study was conducted by taking into consideration the Romanian legal frameworks (law no. 95/2006 on Health Reform, art. 165 and Medical Deontology Code, articles 42–45). The harvesting protocol of this study was evaluated and approved by the Ethics Committee Board of Iuliu Hatieganu University of Medicine and Pharmacy, Cluj-Napoca, Romania, no. 96/18.04.2022.

### 2.2. Study Design and Population

In this observational retrospective study, eight patients from three tertiary (university) centers in Romania, hospitalized in otorhinolaryngology or neurosurgery clinics between November 2021 and June 2022, were included. The patients included in the study were over 18 years old, with a history of COVID-19 confirmed by polymerase chain reaction test (PCR) in the past six months, along with a confirmed histopathological diagnosis of mucormycosis. Patients under the age of 18, without a history of COVID-19 in the previous six months, with chronic rhinosinusitis known/diagnosed in the past year and whose histopathological diagnosis did not confirm mucormycosis were excluded from this study.

Before functional endoscopic sinus surgery (FESS), we performed a preoperative radio-imaging exam in all patients (magnetic resonance imaging (MRI) and/or computerized tomography (CT) scan). During FESS, nine biopsy fragments from the most affected areas of the nasal fossa were collected from each patient: three for histopathological examination, three for scanning electron microscopy (SEM), and three for transmission electron microscopy (TEM), respectively.

### 2.3. Preparation of Samples for the Histopathological Examination

The tissue samples collected during the surgery were fixed in formaldehyde 7% for five days, after which the samples were oriented and placed in cassettes. Tissue processing was performed using a vacuum infiltration processor, namely Tissue-Tek VIP 5 Jr (Sakura, Alphen aan den Rijn, the Netherlands). Paraffin embedding and sectioning were performed using the Tissue-Tek TEC 6 system (Sakura, Alphen aan den Rijn, the Netherlands) and Accu-Cut SRM 200 Rotary Microtome (Sakura, Alphen aan den Rijn, the Netherlands). Slide staining was performed using the Tissue-Tek Prisma Plus automated slide stainer (Sakura, Alphen aan den Rijn, the Netherlands) according to the internal staining protocol using Mayer Modified Hematoxylin (Titolchimica, Rovigo, Italy) and Eosine solution (10 g Eosine B in 1000 mL distilled water). For Gram staining, the Gram Stain Kit (Gram Fuchsin Counterstain) (Atom Scientific, Manchester, England) was used.

For periodic acid Schiff (PAS) staining, tissue sections were treated with 0.5% periodic acid solution for five minutes to oxidize the diol groups in glycogen and other polysaccharides. After incubation, slides were rinsed in distilled water for three minutes. Schiff’s reagent (Sigma-Aldrich, St. Louis, MO, USA) was applied to the sections for 15 min in a dark, humid chamber. The reagent reacted with the aldehyde groups formed by periodic acid oxidation, resulting in the formation of a magenta-colored complex. Slides were washed in running tap water for 10 min to remove excess Schiff’s reagent. Hematoxylin counterstaining was performed for two minutes to visualize nuclei. Slides were then washed, dehydrated through an ethanol series, cleared in xylene, and coverslipped with a mounting medium. Positive control slides with known PAS-positive tissue and negative control slides without the application of periodic acid were included to ensure staining accuracy. Microscopic examination was performed using a Leica DM1000 clinical microscope (Leica, Wetzlar, Germany) with dedicated image acquisition camera and software. All sections were examined by the same investigator (B.G.).

### 2.4. Preparation of Samples for Electron Microscopy

For SEM, the samples collected during the surgery were fixed in 2.7% glutaraldehyde (Electron Microscopy Sciences, Hatfield, PA, USA) for two hours, washed with phosphate-buffered saline and then with distilled water, and were then left to dry. The dried samples were glued to a support with silver paste and sputter-coated with a 10 nm thick Au layer before imaging (Agar Auto Sputter Coater, Agar Scientific Ltd., Stansted, Essex, UK). Scanning electron microscopy (SEM) was conducted on a Hitachi SU8230 cold field emission gun (Tokyo, Japan) at 30 kV. All samples were examined by the same experienced investigator (L.B.T.).

For transmission electron microscopy (TEM), the samples, also collected during the surgery, were fixed in 2.7% glutaraldehyde (Electron Microscopy Sciences) in 0.1 M PBS for two hours, rinsed three times with 0.15 M PBS for one hour each, and postfixed in 2% osmium tetroxide (Sigma-Aldrich, St. Louis, MO, USA) in 0.15 M phosphate buffer. Dehydration was accomplished in an acetone series (30, 50, 70, 80, 90, and three times at 100%). Inclusion was made in EMBed-812 epoxy resin (Electron Microscopy Sciences), which was polymerized for two days at 60 °C. Ultrathin sections of about 80 nm were cut on a Bromma 8800 ULTRATOME III ultramicrotome (LKB Produckter AB, Stockholm-Bromma, Sweden) with glass knives. The sections were collected on 300-mesh copper grids covered by a thin layer of Formvar (Electron Microscopy Sciences). The sections were double-contrasted with 13% uranyl acetate (Merck, Billerica, MA, USA) for 15 min and with 2.8% lead citrate (Fluka AG, Buchs, Switzerland) for five min and examined with a Jeol JEM-100CX II transmission electron microscope (Jeol, Tokyo, Japan) equipped with a Mega View G3 camera (emsis, Münster, Germany). All TEM samples were examined by the same investigator (A.F.). 

## 3. Results

The distribution of the patients by age and sex; as well as the duration between the diagnosis of COVID-19 and mucormycosis; the existence of comorbidities, such as diabetes; the history of corticosteroid intake; and the results of the clinical, radio-imaging, histopathological, and electron microscopy examinations are shown in Table 1.

The ages of the eight patients included in the study varied between 30 and 73 years, with five men and three women, respectively. The duration between the diagnosis of COVID-19 and CAM was one month for three patients (37.5%, n = 3/8), two months for two patients (25%, n = 2/8), and over two months for three patients (37.5%, n = 3/8) (median 44 days). Half of the patients (50%, n = 4/8) had decompensated diabetes, and most patients (62.5%, n = 5/8) reported a history of corticosteroid intake during treatment for COVID-19.

**Figure 1:**
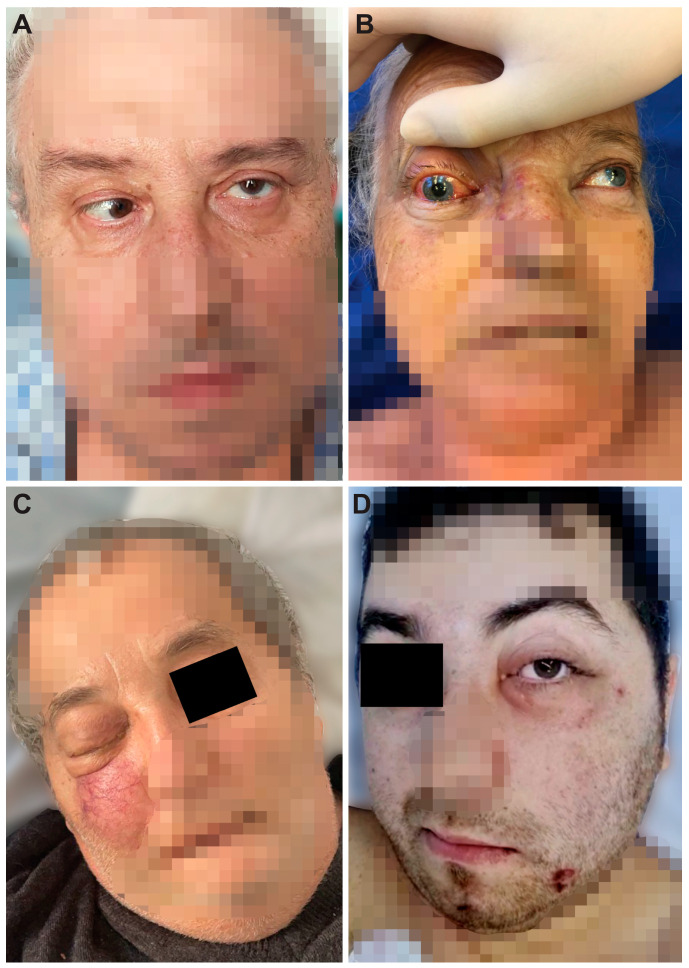
Facies of patients with CAM. (**A**) Immobility of the left eye (the patient looks to the lower left side) and left incomplete blepharoptosis. (**B**) Swelling of the right zygomatic region, exophthalmos with immobility of the right eye (the patient looks to the upper left side), and complete right blepharoptosis. (**C**) Exophthalmos and complete right blepharoptosis. (**D**) Swelling of the left zygomatic region and left incomplete blepharoptosis.

**Figure 2:**
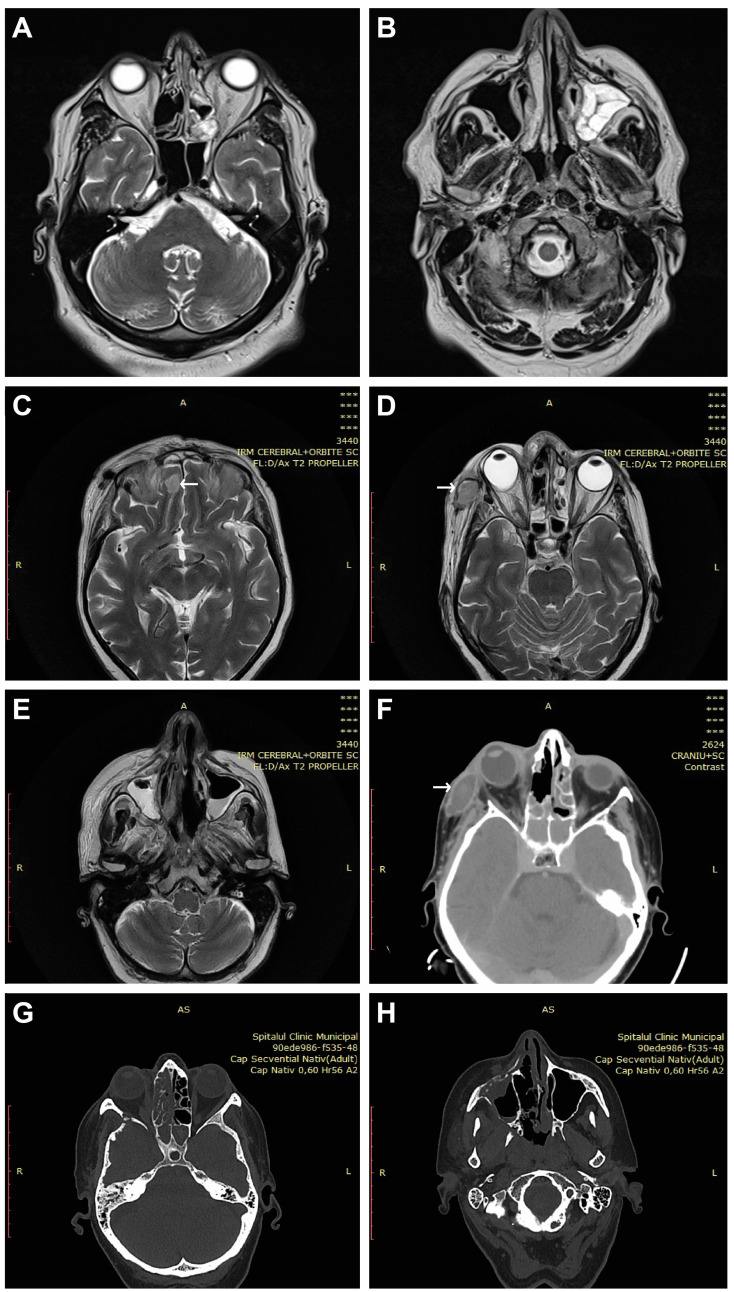
Two CT and MRI radio-imaging aspects in CAM. (**A**,**B**) Left exophthalmos and left maxillo-ethmoidal rhinosinusitis. (**C**) Right paramedian frontal cerebral intraparenchymal abscess (arrow). (**D**–**F**) Altered sphericity of the right eyeball, pansinusitis with inhomogeneous content, and zygomatic abscess (arrows). (**G**,**H**) Right pansinusitis and bone erosions.

**Figure 3:**
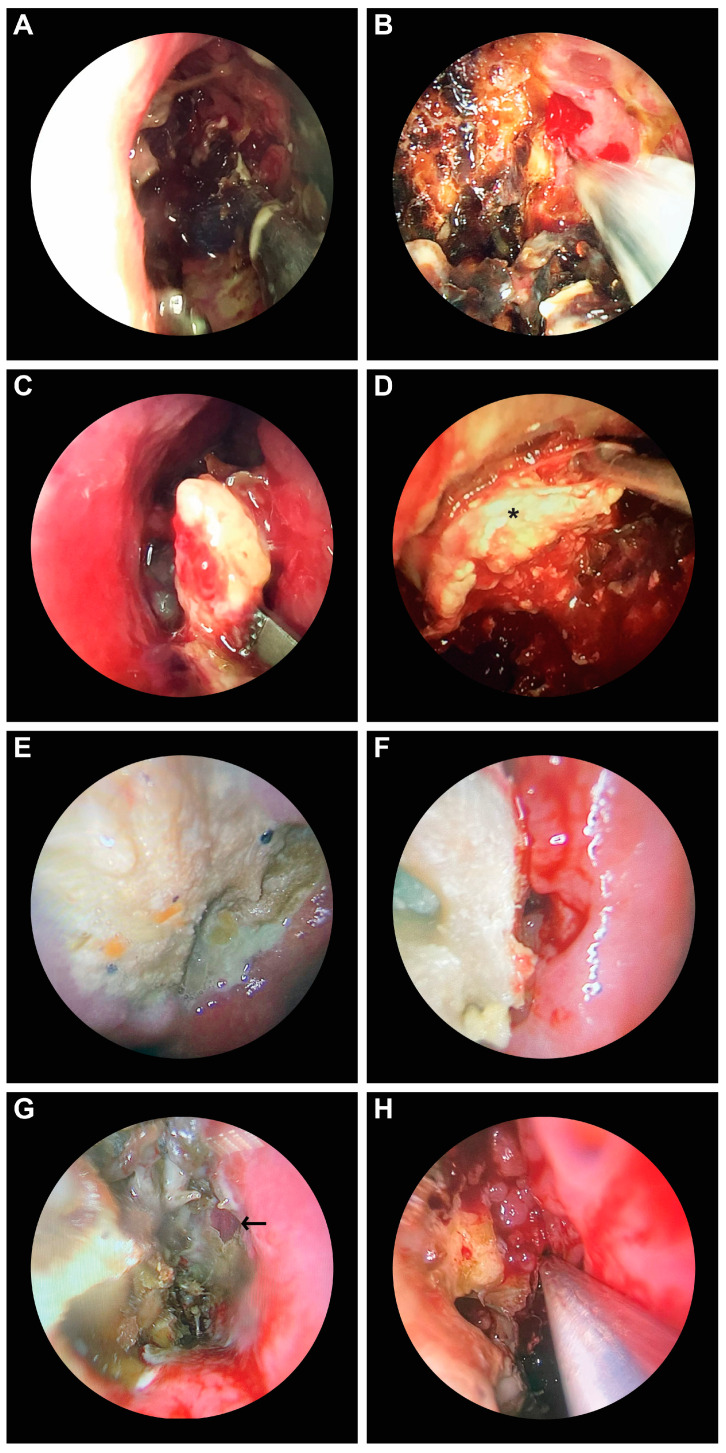

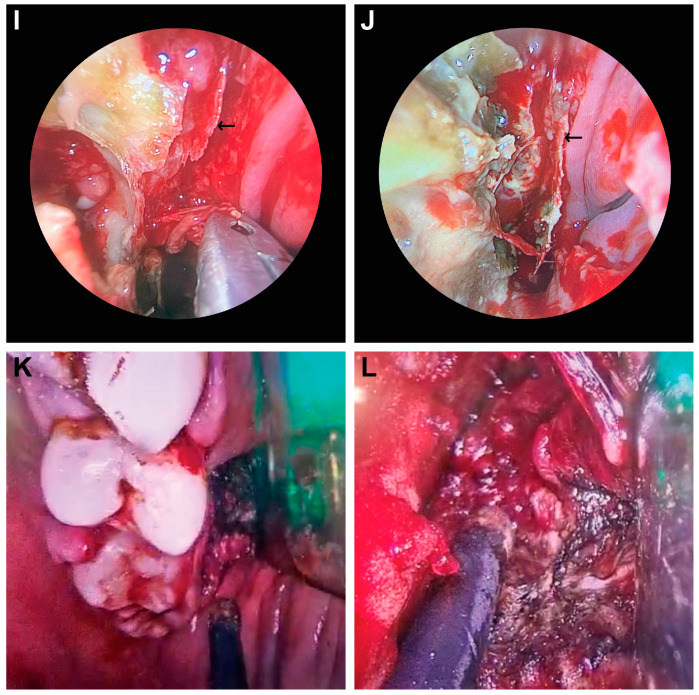
Intraoperative aspects during FESS in CAM. (**A**–**D**) Extensive mucosal necrosis in the right nasal cavity; periorbital fat is highlighted by *. (**E**) Denudation and fungal infiltration of the hard palate; (**F**) oronasal fistula at the junction between the apparently healthy tissue and the infected tissue mass; (**G**) fungal invasion in the right nasal cavity with secondary perforation of the nasal septum (arrow); (**H**) polypoid degenerate rhino-sinusitis; (**I**,**J**) the fungus-infiltrated nasal septum detached in bloc during the operative cure; (**K**) bone erosion at the level of the left alveolar rim and gingival hemorrhage. Surgical exploration needed in order to practice hemostasis. (**L**) Cauterization in the zygomatic region.

The immobility of the eye, exophthalmos, incomplete or complete blepharoptosis, and swelling of the zygomatic region were observed in the facies (Figure 1). Figure 2 shows CT and MRI preoperative radio-imaging aspects, and Figure 3 shows intraoperative aspects during FESS.

Affection of the nasal fossa (Figure 3A–D,G–J) and the homolateral maxillary sinus was observed in all patients included in the study (Figure 2B,E,H). The extension of lesions to all paranasal sinuses on the same side was observed in half of the patients (50%, n = 4/8). The extension of the lesional process at the level of the orbit was observed in 62.5% of patients (n = 5/8) (Figure 1A–D and Figure 2A,D,F), 25% of patients (n = 2/8) presented oral invasion (Figure 3E,F,K), 25% of patients (n = 2/8) presented invasion in the zygomatic region (Figure 1B,D and Figure 2D,F), and one patient (12.5%, n = 1/8) presented cerebral invasion (Figure 2C).

All patients underwent FESS (Figure 3). 

**Figure 4:**
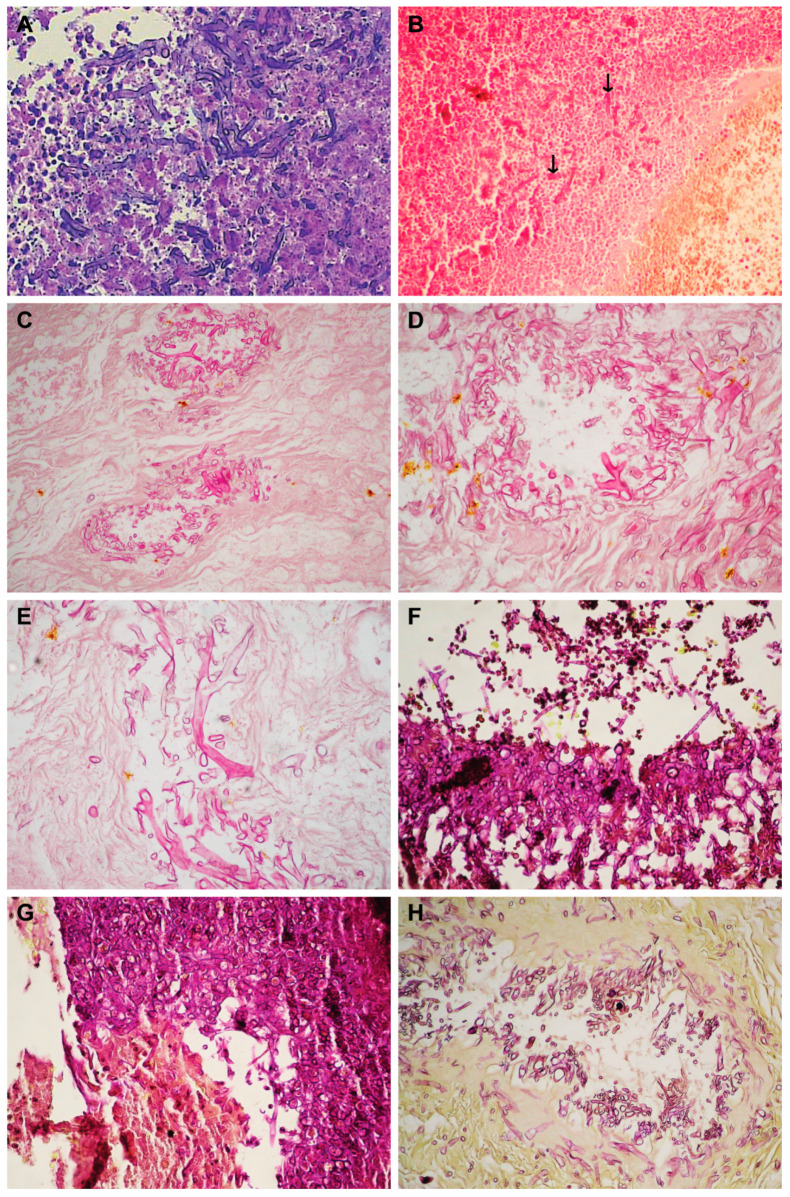

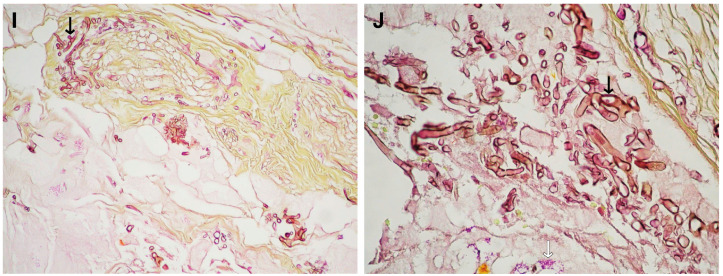
Histopathological aspects of CAM. (**A**) Wide aseptate hyphae, polymorphonuclear cells, necrosis (HE × 40). (**B**) Aseptate hyphae (arrows), angioinvasion, vascular necrosis, hemorrhage (HE × 40). (**C**) Wide aseptate hyphae, angioinvasion (HE × 20). (**D**,**E**) Wide aseptate hyphae, angioinvasion (HE × 40). (**F**,**G**) Septate hyphae suggestive of *Aspergillus* spp. (PAS × 40). (**H**) Wide aseptate hyphae, angioinvasion (PAS × 20). (**I**) Invasion of hyphae in the perineural lymphatics (arrow) (PAS × 20). (**J**) Necrotic area with hyphae (black arrow) and bacterial flora (white arrow) (PAS × 40).

In the histopathological examination of the biopsy fragments collected from the most affected areas of the nasal fossa, the morphological aspects characteristic of mucormycosis were identified in all cases: wide aseptate hyphae with right-angle ramifications, which invade blood vessels (Figure 4A–E,H,J). Only one case presented perineural invasion into the perineural lymphatics (Figure 4I). In only one case, we identified mucormycosis–aspergillosis fungal coinfection (Figure 4F,G). 

**Figure 5:**
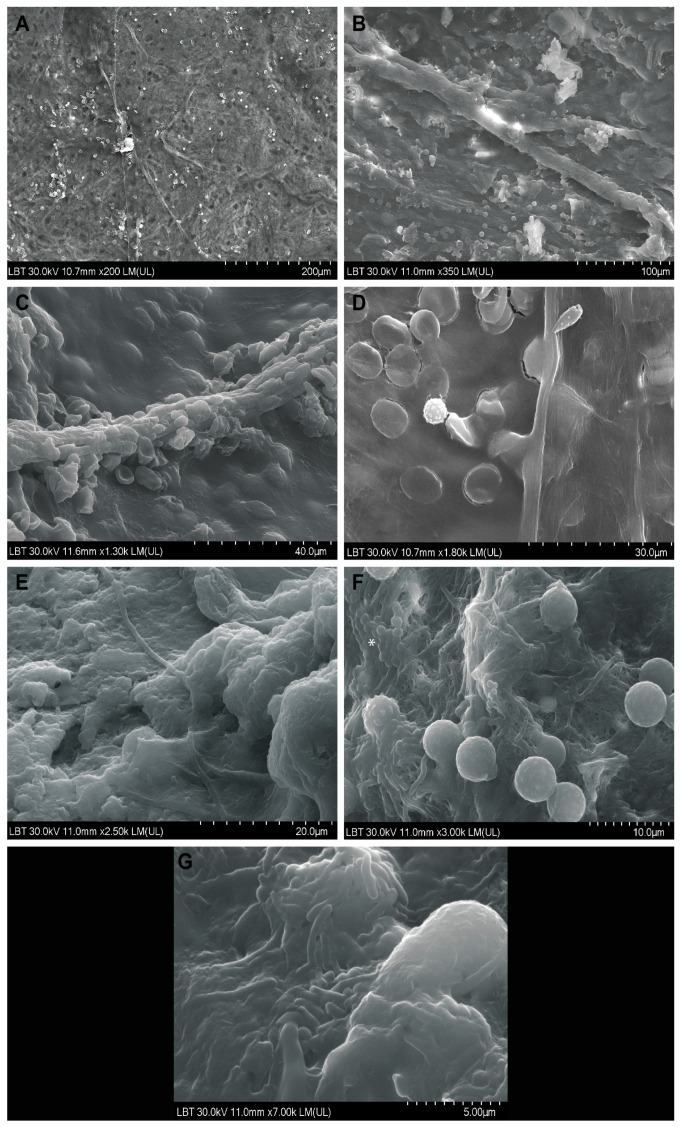
Scanning electron microscopy aspects in CAM: (**A**–**D**) hyphae on the surface of the mucosa, surrounded by cells of the local immune system (**B**,**D**); (**E**) around the hypha, rhizoids can be observed; (**F**) adjacent rhizoids, immune cells, and a biofilm of cocci are observed (asterisk); (**G**) rhizoids and immune cells.

Through SEM, in all analyzed samples, long hyphae were identified on the surface of the mucosa (Figure 5A–D), surrounded by cells belonging to the local immune cells (Figure 5B,D). In half of the samples (50%, n = 4/8), bacterial biofilms were identified (Figure 5F, asterisk), and in three samples (37.5%, n = 3/8), rhizoids were identified (Figure 5E–G).

**Figure 6:**
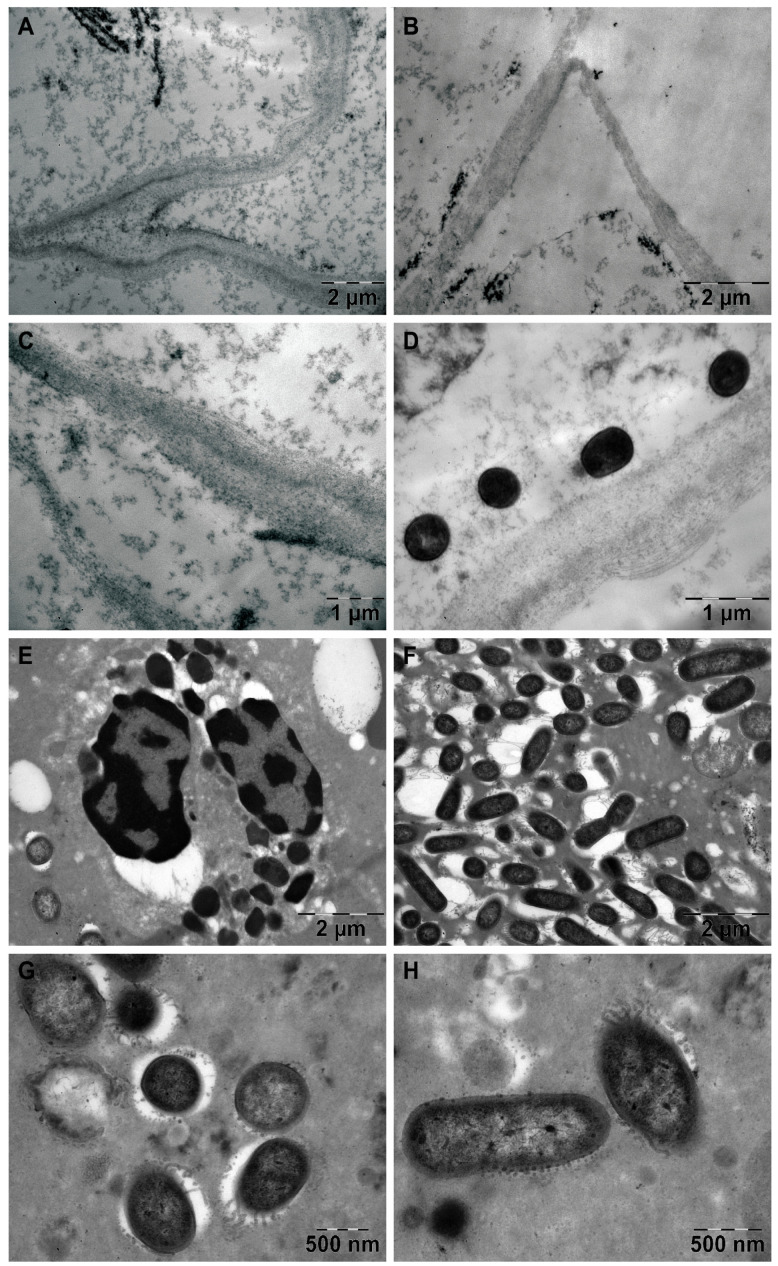
Transmission electron microscopy aspects in CAM: (**A**–**C**) aseptate hyphae; (**D**) cocci located in the vicinity of the hyphae; (**E**) neutrophil with phagocytosis vesicles; (**F**) bacterial biofilm; (**G**,**H**) morphological details of the bacteria in the biofilm.

Through TEM, aseptate hyphae were identified in 87.5% of the samples (n = 7/8) (Figure 6A–D), accompanied by bacterial elements (62.5%, n = 5/8) (Figure 6D–H).

## 4. Discussion

The correlation between COVID-19 and mucormycosis is based, on the one hand, on the changes induced by SARS-CoV-2 on the immune system (immune dysregulation [22]), and on the other hand, on the use of steroids in the treatment of COVID-19 [23], which further suppresses the immune response. Thus, corticosteroids and diabetes mellitus are the most important predisposing factors in the development of CAM [8,9,12,13], which is a fact that can also be observed in our study. 

More than half of the patients included in our study had diabetes, predominantly in a decompensated form. Diabetes has long been established as the main predisposing factor for all types of mucormycosis, with one meta-analysis of 851 cases reporting it in 40% of patients with mucormycosis [2]. In uncontrolled diabetes, ketoacidosis is considered a key factor enabling mucormycosis, with two parallel mechanisms proposed: a low plasma pH reduces the phagocytic effect of macrophages and the oxidative burst of neutrophils, and at the same time, a lower pH reduces the affinity of transferrin for free iron, making metal available to the infecting pathogen [24]. Also, the main comorbidity for mucormycosis in India remained diabetes, as it was found in 54–76% of patients pre-COVID-19 and in 60–92% of patients with COVID-19-associated mucormycosis once the pandemic started [25].

Human and animal model studies have concluded that, in addition to diabetes, being immunocompromised is another key factor that makes individuals more susceptible to mucormycosis [6,26,27]. Long before the COVID-19 pandemic, corticosteroids were recognized as a risk factor for opportunistic fungal infections like mucormycosis due to their complex immunosuppressant effects [28]. A 2021 multicenter study in India, the country with the largest global mucormycosis burden, estimated that the prevalence of mucormycosis in the country doubled in the first year of the COVID-19 pandemic (2020) compared to the previous one, at least in part due to the improper use of corticosteroids in patients with COVID-19. In that study, 78% of 187 patients with COVID-19-associated mucormycosis had received glucocorticoids for their COVID-19 illness compared to just 6% of patients with non-COVID-19-associated mucormycosis [29]. 

The duration between the diagnosis of COVID-19 and CAM seems to have an interval that varies quite a lot, starting from 10–15 days [11,13] to 3 months [30]. In our study, we observed a duration of one-two months (median 44 days).

The clinical aspects vary depending on the extension of the lesional process. With the invasion of bone structures, oroantral [8] and sinus alveolar fistulas [9] appear, as can be observed in cases no. 5 and 8, respectively, in Table 1.

An assessment of the extent of the lesions requires radio-imaging investigations. It is recommended that the diagnostic assessment is not based on CT imaging alone, as MRI resolution is much more sensitive to the early and accurate detection of invasion into non-bony structures [10]. However, we believe that the lack of technical equipment necessary to perform MRI should not delay the initiation of treatment when the clinical suspicion is high, or when the histopathological diagnosis is positive. In the context of the COVID-19 pandemic, the use of ultrasound for paranasal sinus imaging can be considered [31].

A histopathological examination provides certainty regarding the diagnosis. The lack of microscopic identification of hyphae should not lead the physician away from the diagnosis of mucormycosis when the presence of risk factors and the clinical examination suggest the presence of this infection [14]. Histopathological characteristics of mucormycosis are represented by the infiltration of the fungus into the mucosa, sub mucosa, and blood vessels; thrombosis; tissue infarction; and hemorrhage [32]. Vascular invasion and neurotropism are considered common pathogenic features of invasive mucormycosis. The microscopic detection of aseptate or pauci-septate ribbon-like hyphae with a large diameter (hyphae wider than *Aspergillus* spp.) with wide branching angles (90° angles, unlike *Aspergillus* spp. showing septate hyphae that branch at angles of 45°—Figure 4F,G) and irregular branching in tissue is suggestive of mucormycosis [19,33,34,35]. Mucormycosis and aspergillosis coinfection in the oroantral region has been previously reported [36,37,38], including in patients with COVID-19 infection [39]. In our study, we identified this coinfection in case no. 7 in Table 1.

Perineural invasion is a common finding in invasive zygomycosis (Figure 4I). Prior to excluding the suspected presence of these fungi in biopsies, the perineural space should be carefully examined [40]. However, it is quite possible that many biopsies do not contain nerve elements. A marked inflammatory response is often observed in the histopathology of mucormycosis [40]. In all of the samples we studied, we identified polymorphonuclear cells (Figure 4A). Polymorphonuclear neutrophils are responsible for phagocytosis and the destruction of fungi. The acidic environment (found especially in patients with diabetes due to ketoacidosis) allows the fungi to transform into hyphae at the tissue level, and then they invade the blood vessels. This extensive angioinvasion leads to vascular thrombosis, followed by tissue necrosis [14,19]. An extensive histopathology study on 200 patients with post-COVID-19 mucormycosis, carried out in India, showed that angioinvasion was present in 48 patients, perineural invasion was present in 32 patients, and necrosis was present in 121 patients. Invasion was directly proportional to the mortality rate [41]. 

Although in some studies, the diagnosis of mucormycosis was based on fungal culture [8], the role of mycological laboratory examination has little utility in practice as far as rhino-orbital-cerebral mucormycosis is concerned. It is not unusual that tissue samples are not sent for culture because the growth of these types of fungi requires a long period, or these fungi do not grow [1].

Electron microscopy has not been used in the diagnosis of human mucormycosis. A single electron microscopy study specific to human mucormycosis was found, describing macrophage-engulfed hyphae in the cornea of a patient with corneal mucormycosis based on TEM images [42]. Another TEM study described the early post-infection localization and germination of spores in the lung epithelium in murine mucormycosis and aspergillosis models [26]. One case report employed SEM to describe the aspect of *Rhizopus arrhizus* spores collected from a colony grown from a biopsy sample from a patient with rhino-orbital-cerebral mucormycosis [43]. SEM images of spores can be used to distinguish some fungal species (while others have too similar-looking spores), but for reliable identification, a PCR test would be required [44]. Moreover, one cannot precisely identify a specific fungal species based on the morphological characters observed by SEM/TEM when analyzing tissue samples. 

Although, as far as mucormycosis is concerned, from a clinical point of view, electron microscopy has a limited value; it has high experimental significance for patho-physiological understanding. During the adaptation process, microorganisms can change their morphology, including their sizes, branching angles of hyphae, etc. On the other hand, SEM helps us understand the morphology of the nasal sinus microhabitat, sinus dysbiosis, and the morphological bases of the interaction between the microorganism and the cells of the local immune system (see previous studies [45,46]), highlighting the fact that the way in which the infection develops depends on the interaction of the microorganisms with the cells of the immune system (the microbial aggressiveness-immune competence ratio). In our study, bacterial superinfection could be identified by SEM in half of the patients who developed bacterial biofilms in the vicinity of hyphae or rhizoids (Figure 5F). 

TEM shows us the ultrastructure of the hyphae, the associated bacteria, and the morphology of the interaction between them. Through TEM, we identified bacteria in more samples than through SEM, but we were not able to argue for the presence of microbial biofilm because bacteria can detach from the biofilm during sample preparation and can be visualized independently of the biofilm, and the biofilm may not be present in the selected sections. For electron microscopy to provide a correct diagnosis of rhino-orbital-cerebral mucormycosis, the dominant/overall morphological aspect must be considered.

This study has some limitations. First, this study had a small number of patients who met the study’s inclusion criteria. Second, there was a lack of fungal etiological diagnoses via mycological cultures and a lack of identification of the genera and species of the zygomycetes.

## 5. Conclusions

Patients are prone to secondary infections in the post-COVID-19 period. CAM occurs more frequently in patients with diabetes and/or those with a history of corticosteroid intake during COVID-19 infection. An awareness of the existence of CAM, the rigor of clinical examination, and diagnostic suspicion for cases at risk contribute to the early identification of a correct diagnosis.

The histopathological examination highlights the typical morphological characteristics of the fungi in the *Mucorales* ordinal, angioinvasion, and perineural invasion and fungi co-infection. Electron microscopy shows us that infection development depends on the interaction of the microorganisms with the cells of the immune system. Nasal sinus dysbiosis can be present and favors the appearance of bacterial biofilms. 

## Figures and Tables

**Table 1:** Patients’ distribution and clinical and imagistic results of microscopic analysis.

No./Gender/Age	University Center	Duration between the Diagnosis of COVID-19 and Mucormycosis	Diabetes	History of Corticosteroid Intake	Clinical Examination	Radio-Imaging Examination	Histopathological (HP) and Electron Microscopy Result
1, 57, M	Bucharest	one month	type 2, newly diagnosed	Yes	Immobility of the left eye, left incomplete blepharoptosis (Figure 1A). Extensive necrosis in the left nostril, adherent black crusts, purulent discharge in the left middle meatus.	MRI: Left proptosis, left maxillo-ethmoid rhinosinusitis (Figure 2A,B).	HP: Aseptate hyphae, polymorphonuclear, necrosis (Figure 4A), angioinvasion. SEM: Hyphae, rhizoids, immune cells. TEM: Aseptate hyphae.
2, 66, F	Cluj-Napoca	six weeks	-	Yes	Swelling of the right zygomatic region, exophthalmos with immobility of the right eye, complete right blepharoptosis (Figure 1B). Extensive mucosal necrosis in the right nostril (Figure 3A–D).	MRI and CT performed at 2-week intervals. MRI: Right paramedian frontal cerebral intraparenchymal abscess (Figure 2C), altered sphericity of the right eyeball, bilateral pansinusitis with inhomogeneous content (Figure 2D,E), zygomatic abscess (Figure 2D, arrow). CT: Bone erosions in the right nasal cavity, zygomatic abscess (Figure 2F). Extensive lesions compared to previously performed MRI.	HP: Aseptate hyphae, angioinvasion, vascular necrosis, hemorrhage (Figure 4B), polymorphonuclear. SEM: Hyphae (Figure 5A), immune cells. TEM: Aseptate hyphae (Figure 6A,B), bacteria.
3, 64, M	Cluj-Napoca	seven weeks	type 2, compensated	No	Right exophthalmos, immobility of the right eye, right oculomotor paralysis, mucopurulent secretions in the right nostril.	MRI and CT: Right pansinusitis with inhomogeneous content, right intraorbital abscess.	HP: Aseptate hyphae, angioinvasion, polymorphonuclear. SEM: Hyphae, immune cells (Figure 5B–D). TEM: Aseptate hyphae, bacteria.
4, 73, M	Cluj-Napoca	one month	type 2, decompensated	Yes	Mucopurulent secretions in the left nostril.	CT: Left maxillo-ethmoid rhinosinusitis with inhomogeneous content.	HP: Wide aseptate hyphae, angioinvasion (Figure 4C–E), polymorphonuclear. SEM: Hyphae, immune cells, bacterial biofilm. TEM: Aseptate hyphae.
5, 69, F	Timisoara	two months	type 2, decompensated	Yes	Denudation and infiltration of the hard palate (Figure 3E), oronasal fistula (Figure 3F), mucosal necrosis in the right nasal cavity, perforation of the nasal septum (Figure 3G–J).	CT: Right pansinusitis with inhomogeneous content.	HP: Wide aseptate hyphae, angioinvasion, polymorphonuclear. SEM: Hyphae, immune cells, bacterial biofilm. TEM: Aseptate hyphae, bacteria (Figure 6D).
6, 65, F	Timișoara	two months	-	No	Ulcer-necrotic lesions of the right nasal cavity.	CT: Right pansinusitis, bone erosions (Figure 2 G,H).	HP: Wide aseptate hyphae, angioinvasion, polymorphonuclear. SEM: Hyphae, rhizoids (Figure 5E), immune cells. TEM: Aseptate hyphae.
7, 62, M	Timișoara	six weeks	type 2, compensated	Yes	Right exophthalmos, orbital cellulitis, right complete blepharoptosis (Figure 1C). Extensive ulceronecrotic lesions in the right nasal cavity.	CT: Right proptosis, altered sphericity of the right eyeball, right pansinusitis.	HP: Aseptate hyphae, angioinvasion, polymorphonuclear, septate hyphae (Figure 4F,G). SEM: Hyphae, immune cells, bacterial biofilm. TEM: Neutrophils, bacterial biofilm (Figure 6E–H).
8, 30, M	Timișoara	one month	type 1, decompensated	Yes	Exophthalmos, left orbital cellulitis (Figure 1D). Erosion, bone erosion, left superior alveolar rim, gingival hemorrhage (Figure 3K,L). Abnormal tooth mobility. Muco-purulent nasal secretions.	MRI: Left proptosis, left maxillary rhinosinusitis.	HP: Aseptate hyphae, angioinvasion, invasion of perineural lymphatics, necrosis (Figure 4H–J). SEM: Hyphae, rhizoids, immune cells, bacterial biofilm (Figure 5F,G). TEM: Aseptate hyphae, bacteria.

## Data Availability

All of the results are available at the Department of Anatomy and Embryology, Iuliu Hatieganu University of Medicine and Pharmacy, 400006 Cluj-Napoca, Romania. The results of the histopathologic exams are also available at the Department of Head and Neck Surgery and Otorhinolaryngology, University Clinical Hospital of Railway Company, Iuliu Hatieganu University of Medicine and Pharmacy, 400015 Cluj-Napoca, Romania (S.A.); the Department of Ear-Nose-Throat, Victor Babes University of Medicine and Pharmacy Timisoara, 300041 Timisoara, Romania (D.H. and N.B.); and the Department of Ear-Nose-Throat, Carol Davila University of Medicine and Pharmacy, 050472 Bucharest, Romania (M.D. and D.V.). The results of the electron microscopy exams are available at the Department of Cell and Molecular Biology, Iuliu Hatieganu University of Medicine and Pharmacy, 400349 Cluj-Napoca, Romania (A.F.) and at the Electron Microscopy Integrated Laboratory, National Institute for R&D of Isotopic and Molecular Technologies, 400293 Cluj-Napoca, Romania (L.B.-T.).
